# Human Breathomics Database

**DOI:** 10.1093/database/baz139

**Published:** 2020-01-24

**Authors:** Tien-Chueh Kuo, Cheng-En Tan, San-Yuan Wang, Olivia A Lin, Bo-Han Su, Ming-Tsung Hsu, Jessica Lin, Yu-Yen Cheng, Ciao-Sin Chen, Yu-Chieh Yang, Kuo-Hsing Chen, Shu-Wen Lin, Chao-Chi Ho, Ching-Hua Kuo, Yufeng Jane Tseng

**Affiliations:** 1 Graduate Institute of Biomedical Electronics and Bioinformatics, National Taiwan University, No. 1, Sec. 4, Roosevelt Road, Taipei 10617, Taiwan; 2 The Metabolomics Core Laboratory, Centers of Genomic Medicine and Precision Medicine, National Taiwan University, No. 2, Syu-Jhou Road, Taipei 10055, Taiwan; 3 Drug Research Center, College of Pharmacy, College of Medicine, National Taiwan University, No. 33, Linsen S. Road, Taipei 10055, Taiwan; 4 Department of Computer Science and Information Engineering, National Taiwan University, No. 1, Sec. 4, Roosevelt Road, Taipei 10617, Taiwan; 5 Master Program in Clinical Pharmacogenomics and Pharmacoproteomics, College of Pharmacy, Taipei Medical University, No. 250, Wu-Hsing St., Taipei 11031, Taiwan; 6 Genome and Systems Biology Degree Program, National Taiwan University and Academia Sinica, No. 1, Sec. 4, Roosevelt Road, Taipei 10617, Taiwan; 7 Department of Pharmacy, School of Pharmacy, College of Medicine, National Taiwan University, No. 33, Linsen S. Road, Taipei 10055, Taiwan; 8 Department of Obstetrics and Gynecology, National Taiwan University Hospital—Yunlin Branch, No. 579, Sec. 2, Yunlin Road, Douliu, Yunlin County 640, Taiwan; 9 Department of Oncology, National Taiwan University Hospital, National Taiwan University Cancer Center, No. 1, Sec. 4, Roosevelt Road, Taipei 10048, Taiwan; 10 Graduate Institute of Clinical Pharmacy, College of Medicine, National Taiwan University, No. 33, Linsen S. Road, Taipei 10055, Taiwan; 11 Department of Internal Medicine, National Taiwan University Hospital, No. 7, Chung-Shan South Road, Taipei 10002, Taiwan

## Abstract

Breathomics is a special branch of metabolomics that quantifies volatile organic compounds (VOCs) from collected exhaled breath samples. Understanding how breath molecules are related to diseases, mechanisms and pathways identified from experimental analytical measurements is challenging due to the lack of an organized resource describing breath molecules, related references and biomedical information embedded in the literature. To provide breath VOCs, related references and biomedical information, we aim to organize a database composed of manually curated information and automatically extracted biomedical information. First, VOCs-related disease information was manually organized from 207 literature linked to 99 VOCs and known Medical Subject Headings (MeSH) terms. Then an automated text mining algorithm was used to extract biomedical information from this literature. In the end, the manually curated information and auto-extracted biomedical information was combined to form a breath molecule database—the Human Breathomics Database (HBDB). We first manually curated and organized disease information including MeSH term from 207 literatures associated with 99 VOCs. Then, an automatic pipeline of text mining approach was used to collect 2766 literatures and extract biomedical information from breath researches. We combined curated information with automatically extracted biomedical information to assemble a breath molecule database, the HBDB. The HBDB is a database that includes references, VOCs and diseases associated with human breathomics. Most of these VOCs were detected in human breath samples or exhaled breath condensate samples. So far, the database contains a total of 913 VOCs in relation to human exhaled breath researches reported in 2766 publications. The HBDB is the most comprehensive HBDB of VOCs in human exhaled breath to date. It is a useful and organized resource for researchers and clinicians to identify and further investigate potential biomarkers from the breath of patients.

**Database URL**: https://hbdb.cmdm.tw

## Introduction

Breathomics is a branch of metabolomics that quantifies volatile organic compounds (VOCs) collected from human exhaled samples or exhaled breath condensate (EBC) samples using gas chromatography–mass spectrometry and gas sensor-driven electronic nose (eNose). MS-based methods are developed to separate and identify VOCs. Quantification of individual compounds helps to understand the biological mechanisms behind airway diseases (1). The eNose devices are capable of detecting individual or a mixture of molecules, providing results with their probability through the pattern recognition algorithm based on similarity of profiles of exhaled breath (2, 3).

Collected exhaled breath samples contain large amounts of chemical information. Due to its non-invasive nature and ease of collection in clinical settings, exhaled breath samples have gained a lot of research interest in its potential for disease diagnosis. In the past, >3000 VOCs have been detected in the exhaled breath of healthy subjects (4). A total of 1840 VOCs identified from breath, saliva, blood, milk, skin secretion, urine and feces in healthy human subjects are known volatolomes (5). The differences of VOCs between healthy subjects and patients were quantified and found to be associated with diseases. Many of the VOCs in EBC samples are linked to airway disease or lung cancer diagnosis. This is expected, considering that VOCs are transported from organs to the lungs through the circulatory system and exchanged in exhaled breath (6). For example, Phillips *et al.* (7) analyzed breath VOCs in a non-invasive approach to distinguish hospitalized patients from healthy controls, and reported that benzene derivatives and alkanes such as styrene and decane are associated with tuberculosis, both in mycobacterial *in vitro* culture and in the fuzzy logic breath discriminators. Montuschi *et al.* (8) reported that nitric oxide (NO) and carbon monoxide may reflect airway oxidative stress, which is an important pathophysiology of asthma. Montuschi *et al.* showed an increase in 8-isoprostane concentrations in breath condensate correlates with the oxidative stress level in asthmatic patients. As the concentration of 8-isoprostane in breath condensate increases, the oxidative stress level also increases, indicative of asthma severity. For this reason, 8-isoprostane concentrations in breath condensate may be a potential biomarker for examining asthma severity (8). Other potential biomarkers such as nitrosothiols (RS-NOs), leukotriene B4 and nitrite are linked to chronic obstructive pulmonary disease (COPD) (9–11). RS-NOs and nitrite are formed from endogenous NO, which contributes to nitrosative stress in physiology of the airways and may be involved in pathophysiology of airway inflammation (9). Corradi *et al.* (9) showed exhaled RS-NOs are detectable and increased in EBCs of patients with COPD and asthma. Leukotrienes and prostaglandins as lipid mediators in inflammation may play important roles in COPD. Leukotriene B4 may be involved in the recruitment of inflammatory cells in the airways and in oxidative stress (12). Prostaglandin E2 (PGE2) and 8-isoprostane are related to lung cancer (13). During lung carcinogenesis, 5-lipoxygenase and cyclooxygenase 2 (COX-2) are overexpressed. PGE2 and 8-isoprostane as the end products of COX-2 metabolism are increased in airway lumen of lung cancer patients and have been linked to tumor development (13). Disease diagnosis through VOCs in exhaled breath sample is a non-invasive method with great potential because samples of exhaled breath are easily accessible VOC sources to be analyzed (14). Moreover, VOCs patterns can also be used to detect other diseases. Nakhleh *et al.* (15) reported that the VOCs profiles acquired by a nanoarray system are associated with certain diseases, and the pattern of VOCs differs from one disease to another. Other than oncology (15–23) and respiratory medicine (15, 24–26), VOCs such as naphthalene, 1-methyl-, 3-heptanone, methylcyclododecane, 1-alanine ethylamide, (S)-, guanidine, N,N-dimethyl- and hexanal measurement in exhaled breath samples were used to detect diseases in infectiology (24, 26, 27) and neurological diseases (15, 28, 29) as well. The rapid and non-invasive method of collecting exhaled breath has been increasingly considered for disease diagnosis (16, 17, 25, 26, 30–32).

The current practice without the Human Breathomics Database (HBDB) is time-consuming and inefficient. It involves identifying compounds in exhaled breath samples with an analytical platform, manually searching for breathomics data using compound names as keywords in biomedical literature. Researchers may need to search for the compound name in combination with the disease name to retrieve relevant information on diseases and compounds. This process is time-consuming and often results in references with irrelevant information. HBDB aims to curate known human breathomics information into a single, cross-referenced and organized database, providing a reliable platform for keyword searches and browsing in alphabetical order. HBDB uses an automated pipeline to retrieve human breath references and to extract related compounds and diseases information. To date, HBDB includes 2766 human breathomics references, 913 compounds and 49 diseases. Descriptions of breath-related compounds and disease summaries are presented in the following separate tabs: compound, disease and reference views for organized information on each category. With HBDB, the labor-intensive process of manually collecting information on human exhaled breath for biomarker studies can be simplified and accelerated.

## Materials and Methods

### Manual curation of 207 references related to human breath studies

Reference lists were first collected manually by searching ‘volatile organic compound’ or ‘exhaled breath condensate’ with the filter of ‘human’ species from the NCBI PubMed database on 3 November 2015. A total of 975 references with full text were downloaded for further manual reference selection. Medical Subject Headings (MeSH) associated with these references were also collected from the PubMed database. The selection criteria are the following: (i) only English articles were included and non-English articles were excluded, (ii) references with small molecules and diseases were included and (iii) references with clear experiment-control design such as comparison of asthma and control groups were included.

According to the aforementioned criteria, a total of 207 references were selected and read by the researchers in our institute. All compounds and diseases discussed in the 207 references were identified and converted to corresponding PubChem Compound IDs (CIDs) and MeSH IDs. The compound disease reference information was extracted and curated manually. All extracted information of compounds, diseases and references were reviewed twice to ensure the data integrity.

### Automatic extraction of related biomedical terms with VOCs from 2766 human breath literatures

A list of 874 VOCs identified from the exhaled breath of healthy human was collected from the research of De Lacy Costello *et al.* (5). We mapped the 874 breath VOCs to corresponding PubChem CIDs. Through matching PubChem CIDs, we merged and removed duplicates with 99 compounds that were manually collected and curated from literatures in the previous section. A total of 913 unique compounds were stored in the HBDB. Then we used NCBI E-Utilities API to identify compounds from PubChem database and cross-referenced them with those in the PubMed database to download all references corresponding to the 913 compounds in November 2018.

To ensure only human-related references were downloaded, we made sure the MeSH term for humans (MeSH: D006801) is assigned to every reference through the NCBI E-Utilities API. To screen for breath-related references, we retained references with keywords such as ‘breath’ or ‘exhaled’ appeared in the Abstract.

After collecting full text or abstract of related references of VOCs, we removed uninformative paragraph and performed tokenization of full text with Maxent sentence detector in the Apache openNLP library. SemRep was used to perform named entity recognition in the tokenized sentences (33). SemRep is based on Unified Medical Language System (UMLS), developed and maintained by the US National Library of Medicine, integrating MeSH and other useful biomedical resources. If SemRep recognized the named entities (abnormalities, genes, proteins, chemicals or functions) and the alias names of target VOCs in the same sentence, then it is established that the named entities are related to target VOCs.

The recognized named entities are classified to different biomedical information according to the semantic types defined in SemRep. For example, ‘asthma’ is listed under the semantic type ‘disease and syndrome’; therefore, it is identified as a disease. Furthermore, the annotations of UMLS revealed that ‘asthma’ is linked to MeSH of D001249. For this reason, we were able to identify the target VOC with associated disease through MeSH. The related biomedical terms with the target VOCs were extracted and stored into database (34).

Taken together, a total of 913 compounds and 60 diseases, taken from 2766 references, resulting in 910 553 extracted terms and millions of relationships between any two entities were stored in an Object-Relational Mapping framework.

The HBDB web application was constructed using the Rails framework (version 5.0.2) with Ruby (version 2.4.0). The website is hosted by Apache2 (version 2.4). The database is hosted by a MySQL server (version 5.5).

## Results

### Database overview

**Figure 1:**
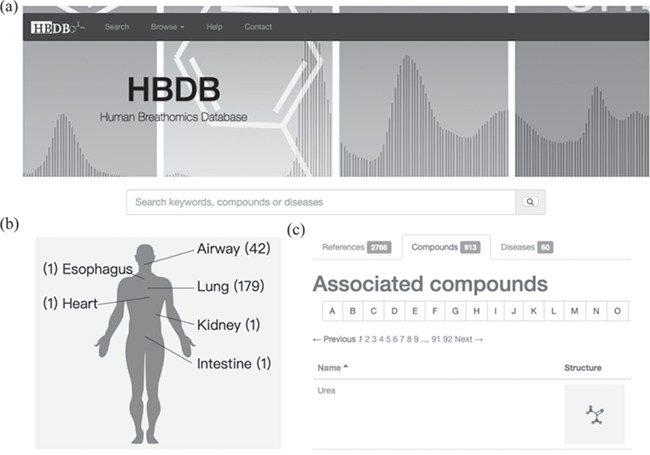
User interface and visualization of disease mapping. (**a**) Browse and search interface of the HBDB. (**b**) Disease mapping to human physiology and statistics of references associated with diseases. (**c**) The collected compounds, references and diseases are listed in three tabs for browsing.

The HBDB is a reference database that includes 2766 references, 913 compounds and 60 diseases associated with human breathomics. The HBDB is designed to provide organized information on references, compounds, diseases and extracted biomedical information from related literatures. Users can find references and compounds with the browse and search panel on the home page (Figure 1a). Diseases mapped to human physiological models with their associated reference counts are also provided on the home page (Figure 1b) and the disease page. The collected compounds, references and diseases are displayed in three tabs (Figure 1c).

We have created separate display views to access the content of the HBDB for references, compounds and diseases. Researchers can search associated compounds, diseases and references by keywords. For example, using ‘asthma’ as the keyword returns 419 references, 18 compounds and 8 diseases in HBDB.

### Compound view

In the compound view, researchers can retrieve summaries of all diseases associated with the compound and downloadable lists of associated references and diseases with external database identifiers, such as PubMed IDs and MeSH terms. The summaries of associated diseases were curated and organized by researchers at our institute. The discussed mechanisms of diseases associated with compounds are described in the summaries. The summaries also provide quantification levels of the compound with associated diseases as described in the references. With the help of text mining full text of associated references, we extracted associated biomedical information (terms) in aspects of abnormalities, chemicals, functions and genes/proteins. The summaries of related biomedical information are composed of extracted terms with highest counts of sentences.

In addition, all associated references of the compound are listed in ascending order by publication year. The reference title links to the reference view in HBDB. Associated diseases are also provided in alphabetical order. The physiological mapping of all associated diseases is displayed in red on a human physiology map. The table of associated diseases consists of disease names linked to the disease view in HBDB, a description of the disease from the MeSH database and associated citations relevant to the disease.

The extracted terms are organized in tables of abnormalities, chemicals, functions and genes/proteins. Each table provides the matched concept name (defined name by UMLS), external id with link to corresponding database, weighted score (range from 0 to 1000, higher score stands for better matched) (34), number of matched sentences (hits) and reference list.

### Disease view

In the disease view, disease description collected from the MeSH database is used for annotations of the disease. The associated references are listed in ascending order by publication year with their title, journal, authors and PubMed ID. The total number of associated references is displayed inside the green box next to the ‘References’ heading in the disease view. The associated references can be readily downloaded by clicking the download button under the ‘References’ heading. The detected compounds in the associated references are also listed in the table in alphabetical order. The PubChem CIDs and external links to PubChem are provided in the table of associated compounds. The total number of associated compounds and a download button for all associated compounds are located in the ‘Compounds’ heading in the disease view. The name of each compound leads to the compound view in HBDB.

### Reference view

In the reference view, authors and citation information with abstracts are provided. All associated compounds reported in this reference are displayed in alphabetical order. Each associated compound is linked to the compound page through the hyperlink on the compound name.

### Database update frequency

Related references of VOCs and the extracted biomedical information are updated monthly. The data of UMLS are updated annually.

## Using HBDB

**Figure 2:**
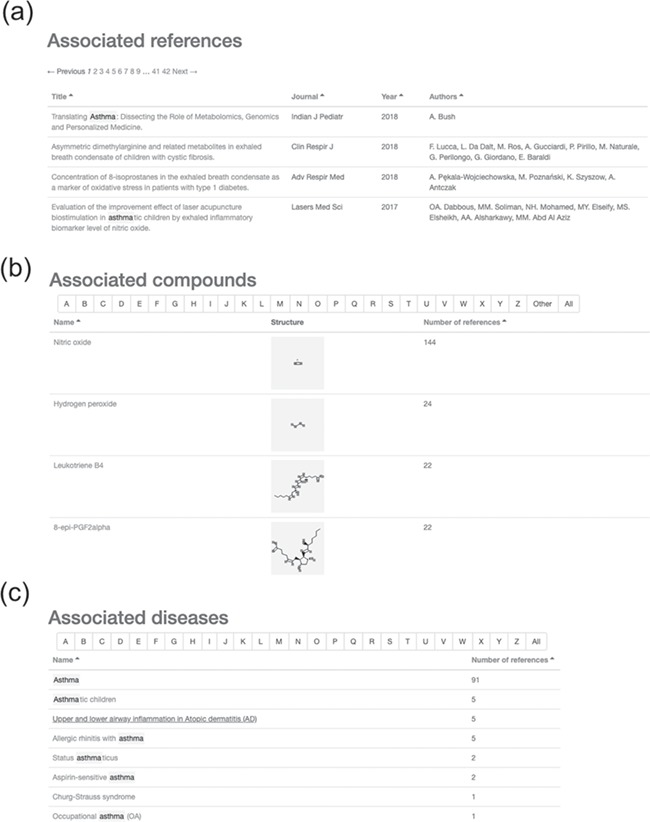
Results of searching for ‘asthma’ in the HBDB. (**a**) Associated references for asthma. (**b**) Associated compounds for asthma in descending order of related number of references. (**c**) Associated diseases for asthma.

We demonstrate how to use the HBDB for finding associated diseases, compounds and references by selecting ‘asthma’ and ‘chronic obstructive pulmonary disease’ as keywords for two cases to display the organized information in HBDB. When ‘asthma’ is queried in HBDB, 419 associated references, 18 compounds and 8 diseases are displayed in tabs of compounds, references and diseases (Figure 1c and details in Figure 2). The search results of associated references contain the title, journal name, published year and authors (Figure 2a). The associated compounds are sorted in descending of the associated reference counts in the table (Figure 2b). Each row in the compound table contains the compound name with a chemical structure. NO, 8-epi-prostagladin F2alpha (8-epi-PGF2α) and leukotriene B4 are the most documented compounds associated with asthma in HBDB. The diseases with the queried keyword ‘asthma’ are listed in Figure 2c, such as asthma, asthmatic children and allergic rhinitis with asthma. Each disease name contains a link to a disease page. Users can determine which diseases may be associated with the queried keywords in the disease table.

**Figure 3:**
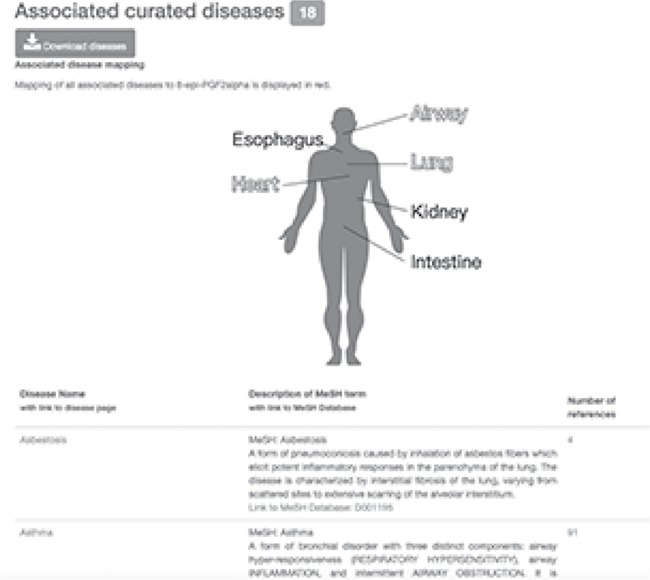
Information on 8-epi-PGF2α with associated curated diseases. Diseases associated with 8-epi-PGF2α are mapped to a human physiological map in red and listed in a table with MeSH definition.

**Figure 4:**
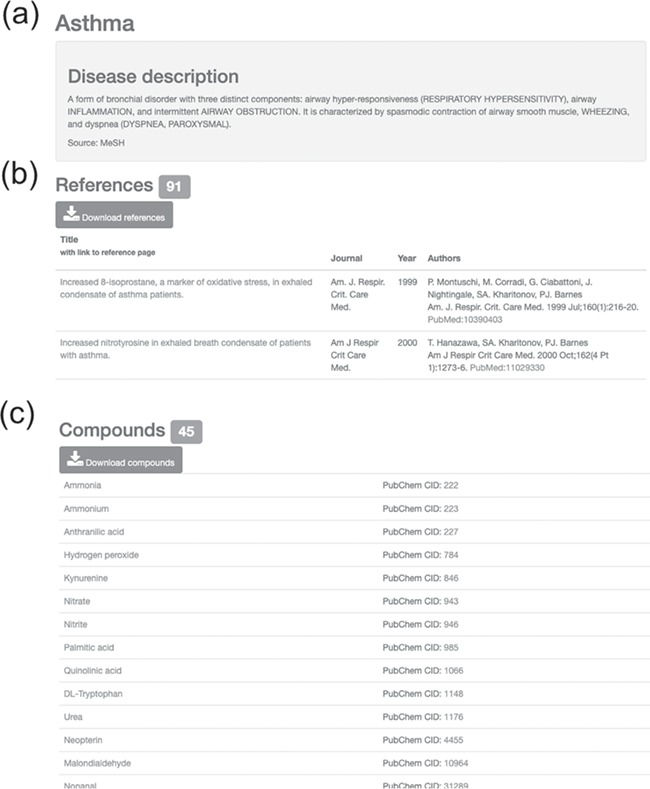
Information on asthma with associated references and compounds. (**a**) Disease description. (**b**) Downloadable list of associated references for asthma. (**c**) Downloadable list of associated compounds for asthma.

In the compound view, users can view information on possible mechanisms in the disease based on the descriptions from collected references and can understand how the compound is linked to asthma or other diseases. 8-epi-PGF2α, also known as 8-isoprostane, is a marker of oxidative stress in EBC of asthmatic patients (8). 8-Isoprostane is a stable, endogenous and biologically active compound, making it a promising marker of oxidative stress in asthma, COPD or respiratory inflammation diseases (8, 35). Increased concentrations of 8-isoprostane are reported in subjects with asthma, COPD, obstructive sleep apnea, airway inflammation and pulmonary diseases (8, 35–37). Diseases associated with 8-isoprostane are mapped and visualized on a human physiological map (Figure 3). Users can see that 8-isoprostane is associated with airway, lung and heart diseases, such as asthma, lung cancer and heart failure, by viewing the organized disease list in the compound view of the HBDB (8, 38). Users can also read the list of associated references in the disease view of the HBDB (Figure 4).

As 8-isoprostane is a marker of oxidative stress in compounds associated with asthma, 3-nitrotyrosine is a product of reactive oxidative species, which leads to damaged cells. 3-Nitrotyrosine is increased in the EBCs of asthmatic patients and is considered a marker of oxidative stress in asthma (39). In addition to correlating with asthma, 3-nitrotyrosine is also correlated with cystic fibrosis (CF). One study reported significantly increased levels of nitrotyrosine in CF compared with normal subjects. The elevation in nitrotyrosine reflects increased oxidative stress in CF patients (40). Another study reported that free 3-nitrotyrosine failed as a marker of oxidative stress in EBCs of children with asthma and CF (41).

Extracted biomedical terms associated with 8-isoprostane also provide information. From the extracted abnormalities table, we could know obesity may be associated with 8-isoprostane. Obesity is associated with systemic inflammation and increased oxidative stress (42). In the work of Komakula *et al.* (43), body mass index is correlated with increased level of exhaled 8-isoprostane in asthmatic subjects. In the work of Ali and Ulrik, authors inferred obesity subjects may have airway oxidative stress and asthmatic subjects may have systemic oxidative stress (44).

**Figure 5:**
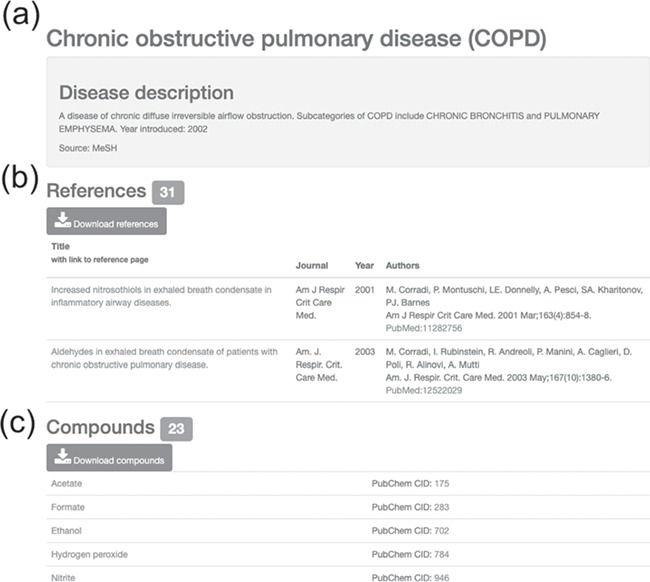
Information on COPD with associated references and compounds. (**a**) Disease description. (**b**) Downloadable list of associated references for COPD. (**c**) Downloadable list of associated compounds for COPD.

When searching for COPD in HBDB, 34 associated references and 27 compounds are displayed on the disease page of COPD (Figure 5). Acetate, one of the compounds listed in association with COPD, is an important compound in pyruvate metabolism, carbon metabolism and beta-oxidation of fatty acids. From the organized description on the acetate page in the HBDB, we can see that acetate is correlated with COPD, pulmonary Langerhans cell histiocytosis (PLCH) and CF. In the compound view of acetate, the associated reference list and associated diseases are listed below the description of acetate. From the associated reference list of the compound view of acetate, we can review the first article via PubMed database by clicking the PubMed link of the first associated reference to see that nucleic magnetic resonance (NMR)-based metabolomics was used to detect and quantify acetate. When we review the second associated reference, we can find a smoking-related diseases study that included COPD and PLCH; the concentrations of both propionate and acetate in COPD and PLCH patients were higher than those in healthy smokers (45). Also, the description of acetate is also provided in the compound view of acetate. After reading the description of acetate, we will know increased levels of acetate in both COPD and PLCH patients may be linked to increased levels of beta-oxidation of fatty acids, as this link was reported in A549 human alveolar epithelial carcinoma cells exposed to smoke (46). Moreover, increased acetate may also be correlated with cholesterol metabolism in the formation of cholesterol with acetate units. Furthermore, we will know an increased level of acetate can be linked to anti-inflammatory action through reading the work of Laurentis *et al.* (45).

Short-chain fatty acids including propionate and butyrate can regulate immune system reactions such as the production of cytokines and chemokines toward the inflammatory process of COPD and Langerhans cell recruitment. The beta-oxidation of butyrate, which generates acetate, is reported to increase in smoke-exposed A549 cells, supporting the fact that acetate increases anti-inflammatory responses in COPD and PLCH (45). A difference in acetate concentrations was also observed between CF and healthy controls using NMR (47). Unlike COPD and PLCH patients, acetate concentrations in CF patients were lower than those in the healthy controls. By contrast, ethanol concentrations increased in CF patients compared with healthy controls. Increased ethanol and decreased acetate may be related to the reduced ability of *Pseudomonas aeruginosa* to oxidize ethanol to acetate.

## Limitation

Human breath-related references are curated based on MeSH annotations; therefore, the latest references cannot be incorporated for analysis without their corresponding MeSH annotations. Related references also cannot be incorporated into the HBDB without available full text sources from HTML or XML files. We may not be able to extract related biomedical information from these references using our current pipeline.

## Conclusion

The HBDB is the most comprehensive HBDB of VOCs in human exhaled breath to date. This database manually curated human breath references from available literature to extract compound disease reference information. To keep the HBDB up-to-date, we applied an automated pipeline using a text mining approach to organize information of compounds, references and diseases related to human breathomics. With the help of the HBDB, researchers can retrieve a wide array of information on associated metabolites and references by searching for a disease of interest. HBDB aims to be a powerful resource that researchers and clinicians may rely on to identify and further investigate potential biomarkers from the breath of patients.

## Author contributions

Y.J.T. conceived the project. C.E.T. and J.L. collected literatures. T.C.K., C.E.T., S.Y.W., O.A.L., B.H.S., M.T.H., J.L., Y.Y.C., C.S.C., Y.C.Y., K.H.C., S.W.L., C.C.H. and C.H.K. read literatures and organized summaries of compounds. T.C.K. constructed the text mining pipeline. T.C.K. and C.E.T. designed and implemented the database. T.C.K., S.Y.W., O.A.L. and Y.J.T. wrote the manuscript. All authors reviewed, revised and approved the manuscript for submission. T.C.K., C.E.T. and S.Y.W. have equal contribution to this work.
